# Is belief larger than fact: expectations, optimism and reality for translational stem cell research

**DOI:** 10.1186/1741-7015-10-133

**Published:** 2012-11-06

**Authors:** Tania Bubela, Matthew D Li, Mohamed Hafez, Mark Bieber, Harold Atkins

**Affiliations:** 1 Department of Public Health Sciences, School of Public Health, Edmonton Clinic Health Academy, University of Alberta, Edmonton, Alberta, Canada; 2 The Ottawa Hospital Research Institute, The Ottawa Hospital General Campus, Ottawa, Ontario, Canada

**Keywords:** stem cell therapy, clinical trials, public expectations, newspaper coverage, commercialization, ethics

## Abstract

**Background:**

Stem cell (SC) therapies hold remarkable promise for many diseases, but there is a significant gulf between public expectations and the reality of progress toward clinical application. Public expectations are fueled by stakeholder arguments for research and public funding, coupled with intense media coverage in an ethically charged arena. We examine media representations in light of the expanding global landscape of SC clinical trials, asking what patients may realistically expect by way of timelines for the therapeutic and curative potential of regenerative medicine?

**Methods:**

We built 2 international datasets: (1) 3,404 clinical trials (CT) containing 'stem cell*' from ClinicalTrials.gov and the World Health Organization's International Clinical Trials Registry Search Portal; and (2) 13,249 newspaper articles on SC therapies using Factiva.com. We compared word frequencies between the CT descriptions and full-text newspaper articles for the number containing terms for SC type and diseases/conditions. We also developed inclusion and exclusion criteria to identify novel SC CTs, mainly regenerative medicine applications.

**Results:**

Newspaper articles focused on human embryonic SCs and neurological conditions with significant coverage as well of cardiovascular disease and diabetes. In contrast, CTs used primarily hematopoietic SCs, with an increase in CTs using mesenchymal SCs since 2007. The latter dominated our novel classification for CTs, most of which are in phases I and II. From the perspective of the public, expecting therapies for neurological conditions, there is limited activity in what may be considered novel applications of SC therapies.

**Conclusions:**

Given the research, regulatory, and commercialization hurdles to the clinical translation of SC research, it seems likely that patients and political supporters will become disappointed and disillusioned. In this environment, proponents need to make a concerted effort to temper claims. Even though the field is highly promising, it lacks significant private investment and is largely reliant on public support, requiring a more honest acknowledgement of the expected therapeutic benefits and the timelines to achieving them.

## Introduction

Stem cell (SC) therapies hold remarkable promise, particularly in the management of diseases and conditions for which there are currently limited or no treatment options [1]. As with all emerging technologies, creating novel applications from an evolving knowledge base is a slow process. Often development commences in niche areas, and only after initial success does the technology expand to broader use. Many SC investigators, therefore, feel that there is a significant gulf between public expectations and the reality of progress toward clinical SC application; a phenomenon observed for other novel biotechnologies [2]. In this paper, we examine this gulf by comparing public expectations, as represented in media coverage, with translation of SC research towards the clinic, as represented in registers of clinical trials (CT).

This paper examines media coverage in an era of expanding SC clinical activity around the globe. Media coverage both shapes and reflects public expectations for SC research [3]. The media record provides a general timeline of translational SC research and spikes in attention that reflect specific events [4]. As media reports are a major source of health and science information for the public [3], they also represent the public face of SC research and thus the messages on which public expectations are based. Likewise, examining the current clinical trial landscape reveals the reality and direction of ongoing improvements to established SC applications and the development of new applications based upon innovative research. While SC transplants are the standard of care for hematopoietic cancers and are gaining acceptance in the treatment of burns and corneal disorders, pioneering SC therapies directed at the regeneration of other tissues and organs (that is, the promise of regenerative medicine) are few in number, use adult rather than embryonic stem cells, and are in the early stages of clinical investigation.

In this paper, we characterize the gap between public expectations represented in media coverage and the spectrum of ongoing SC clinical trials. We first describe the influences that can alter expectations for SC research. We then describe our methods and results, which focus on the subset of trials that comprise novel regenerative medicine applications and representations of the types of SCs used and diseases to be treated or cured. Our discussion contextualizes the gap between current realities and expectations, concentrating on the scientific, regulatory, ethical, and commercial challenges facing SC research as it progresses through clinical development to clinical application. Our goal is to uncover the nature of the biases in the public discourse on stem cell research that separates fact from fiction. This is the first step to creating better communication strategies among the clinical research community and the public, patients, and policy makers. The latter stakeholders need to understand what realistically to expect by way of timelines for the therapeutic and curative potential of regenerative medicine.

## Background

There are many potential reasons for the public's high expectations for SC based therapy [5]; however, advances in the stem cell arena reported to the public may be skewed by media coverage that focuses preferentially upon ethically charged topics [4]. This could fuel overly optimistic speculation by investigators and patient advocates looking to counter ethical opposition to human embryonic (hESCs) and fetal SC research, even though ethical objections have been tempered with the discovery of induced pluripotent SCs (iPSCs) [6]. Further inflation of expectations occurs through direct advertising to desperate patients by clinics offering unproven SC therapies, which have sprung up in jurisdictions with lax regulatory oversight [7] and by dramatic situations portrayed in popular culture [1,8], such as the growth of multiple organs from SCs on *Grey's Anatomy*.

Inflation of expectations may also result from societal pressures such as: the potential for economic returns on research investment, job creation in a new industry, and the fear that commercial benefits will flow elsewhere in the absence of a supportive funding and regulatory environment [5]. The notion of research, largely from academic centers, as an economic engine has been gaining momentum since the 1960s when policymakers began linking innovation with productivity and economic growth [9]. This argument has fueled support for public funds for biomedical research, but, in the case of SC research, Vannevar Bush's observation may hold that 'a belief may be larger than a fact' [8]. Policy reports claim SC therapies will, in the foreseeable future, not only regenerate damaged tissues and organs, but enable the growth of transplantable organs and combat rising healthcare costs [10]. Policy-based estimates of economic return range over orders of magnitude from tens of millions to billions of dollars [5] while predicted employment gains vary from tens of thousands to hundreds of thousands of jobs. Jurisdictions as disparate as India, China, Japan, Singapore, the UK, Australia and Canada all signal intense international competition for economic advantage, each declaring a bid for pre-eminence. For example, in his parting speech to the Labour Party, former British Prime Minister Tony Blair declared: 'How to be the world's number one place of choice for bioscience: if America does not want stem-cell research, we do' [11]. Yet, contrary to the hype, large-scale corporate investment in the field has been slow to materialize because of the lack of proven business and investment models.

Nevertheless, many researchers attempt to temper expectations and timelines, acknowledging the complexity of cellular processes and the need for a better understanding of developmental biology [1,12]. Based on the history of advances in biotechnology, George Daley has recently stated that, 'it takes some two decades for a new biotechnology advance to translate into widely successful clinical therapies' [12]. Given that hESCs were first described in 1998 and iPSCs in 2007, 'we might anticipate some 10 to 15 years before effective products are developed, thereby launching the era of regenerative medicine' [12]. Elsewhere, however, Daley has stated: 'We should be humble and appreciate it may take us the better part of this century to truly harness the power of cells as medicines' [13].

Regulators are similarly taking a cautionary approach to emerging applications [14,15], despite building pressures to accelerate the development of SC therapies. Regulators share the concerns of many investigators that exaggeration and hype have real-world consequences, currently manifested by the rise in SC tourism [16] to SC clinics [7]. Services are offered most commonly for neurological diseases, including multiple sclerosis, stroke, Parkinson's disease, spinal cord injury and Alzheimer's disease, and cardiovascular diseases [7]. SC tourism has necessitated a response by the International Society for Stem Cell Research (ISSCR) because 'administering unproven SC interventions outside a carefully regulated clinical trial puts individual patients at risk and also jeopardizes the legitimate progress of translational SC research' [14]. These scientific, regulatory and commercial challenges are at odds with public expectations, which seek to increase the pace of clinical development and have healthcare systems fund what will likely be personalized and costly therapies.

## Methods

### Newspaper articles

We built a dataset of newspaper articles using Factiva.com (Dow Jones & Co.), a comprehensive database of major newspapers from the USA and UK, which also includes all major newspapers in Canada. We searched without date restrictions for: ('stem cell' or 'stem cells') AND (therapy or therapies or therapeutic or therapeutics or treatment or treatments). Factiva identified and removed duplicates. We then analyzed both our CT dataset described below (title, brief summary, and detailed description for each CT) and the full-text newspaper dataset, using a custom word frequency analysis program that enabled a set of documents to be searched using multiple words or phrases, separated by and/or. The result of each search query was not the number of times that a search term occurred; rather, it was the number of documents containing the term(s). We compared word frequencies using χ^2 ^analyses in two time blocks: 1990 to 2000 and 2001 to 2010. While the broad search strategy may have captured newspaper articles that were only tangentially related to translational SC research, our keyword analysis extracted newspaper articles from this set that specifically referred to SC types and disease categories.

### Clinical trials

We searched the term 'stem cell*' in the world's largest and most comprehensive database for CTs: ClinicalTrials.gov and a second portal that captures international trials not registered with the US National Institutes of Health (NIH): the World Health Organization's International Clinical Trials Registry Search Portal [17]. The latter included trials from Australia and New Zealand, UK, Brazil, China, India, South Korea, Cuba, Germany, Iran, Japan, Africa, Sri Lanka, and The Netherlands. We removed non-SC-related trials that were nevertheless captured by the search and duplicates (trials registered in more than one database). We constructed a database of CT information using all fields, limiting our analysis to CTs registered before 1 January 2011.

We coded all CTs in the dataset for the principle disease/condition addressed, and CTs could fall within more than one category. We then developed inclusion and exclusion criteria to identify groundbreaking or novel SC therapies and coded the trials based on their description supplemented by related publications and websites. The latter was necessary, for example, to characterize a therapeutic agent identified only by its proprietary name in the trial description.

Our definition of novel SC therapies was CTs that involved: (1) the use of stem or precursor cells to stimulate non-hematopoietic organ regeneration (for example, limbal SCs for corneal regeneration or hematopoietic SCs for cardiac repair); (2) the use of agents to stimulate stem or precursor cell action for regenerative or therapeutic purposes; (3) the use of established hematopoietic SC transplantation procedures for novel indications (for example, autoimmune or congenital diseases); (4) the use of novel agents or processes for stem or precursor cell collection, purification or expansion; and (5) the use of genetically modified stem or precursor cells. These categories fall predominantly within Mason *et al*.'s definition of regenerative medicine [18].

We excluded CTs from our 'novel' category that were observational in nature, for example, some CTs measured circulating endothelial precursor cells without a therapeutic goal; involved an established SC therapy for an established indication, most commonly hematopoietic SC transplantation for hematological malignancies; or investigated supportive measures surrounding an SC therapy, for example, antibiotics to prevent infection in hematopoietic SC transplant recipients.

## Results

The analysis was based on the total of 13,249 newspaper articles collected using our broad search strategy and 3,404 CTs from 1978 to 2011. The apparent decline in CTs from 2009 to 2010 may reflect economic decline from 2008, but is more likely due to a lag in the registration of trials. It is worth noting that there was a slight rise in CTs after 2000, which may be the result of the institution of stricter registration requirements for acceptance of CTs for publication [19].

### Newspaper articles

**Figure 1:**
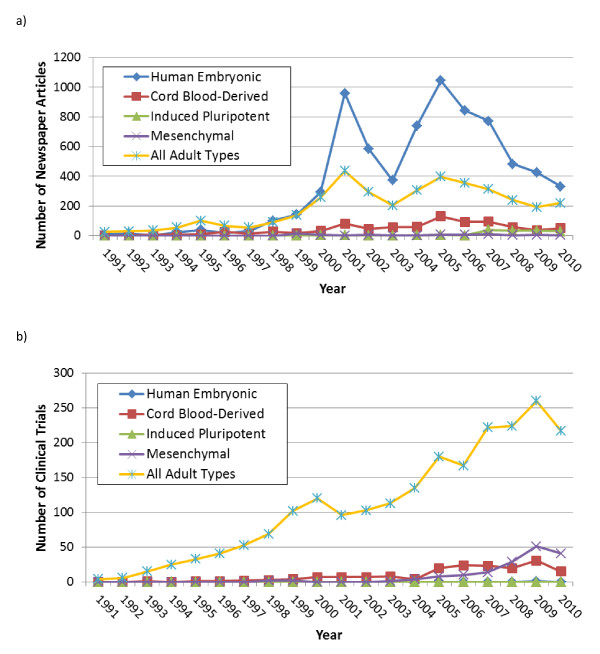
**Stem cell types in newspapers (A) and clinical trials (B) from 1991 to 2010**. The difference in the percentage of newspaper articles versus clinical trials using keyword synonyms for stem cell types is significant overall as well as between the decades 1991 to 2000 and 2001 to 2010 (*P *<0.001).

**Figure 2:**
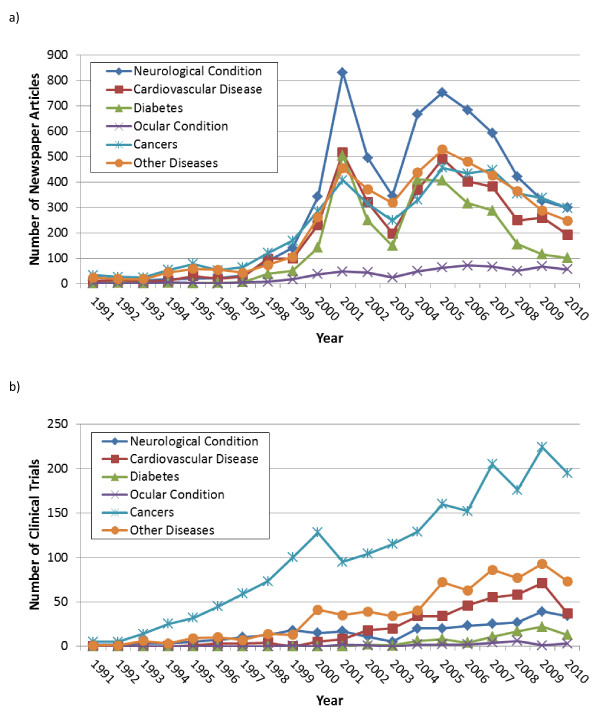
**Categories of diseases in newspapers (A) and clinical trials (B) from 1991 to 2010**. The difference in the percentage of newspaper articles versus clinical trials using keyword synonyms for stem cell types is significant overall as well as between the decades 1991 to 2000 and 2001 to 2010 (*P *<0.001).

Newspaper articles focused mainly on human embryonic SCs and neurological conditions with significant coverage of cardiovascular disease and diabetes as well. Newspaper coverage peaked in 2001 and again in 2005, coinciding with US President George Bush's 2001 Directive limiting the use of federal funds for derivation of new hESC lines and similar discourse following his re-election at the end of 2004 (Figure 1A) [4]. Notably, a similar spike in coverage did not accompany US President Barack Obama's Executive Order, signed 9 March 2009, reversing President Bush's Directive. We discussed the framing of the issues in newspaper coverage around hESC research in a previous paper [4]. The key result here is that hESCs dominated newspaper coverage of SC research, with slight coverage of the discovery of iPSCs in November 2007, mainly focused on their being an 'ethical' alternative to hESCs (Figure 1A) [6]. This leaves hESCs front and center in the public mind with respect to the type of stem cell in clinical development. In contrast, while newspapers referred to adult SCs and occasionally their tissue of origin, they rarely referenced terms such as hematopoietic and mesenchymal, or synonyms for these such as blood SCs. In addition, newspaper articles focused disproportionately on neurological conditions, primarily, multiple sclerosis, stroke, Parkinson's disease, spinal cord injury and Alzheimer's disease with significant coverage also of cardiovascular disease and diabetes (Figure 2A). Neurological diseases were significantly linked to coverage of hESC research and therapies. Promising research in therapies for ocular conditions such as corneal repair and macular degeneration received a small but steady amount of coverage from 2000 onwards.

### Clinical trials

**Figure 3:**
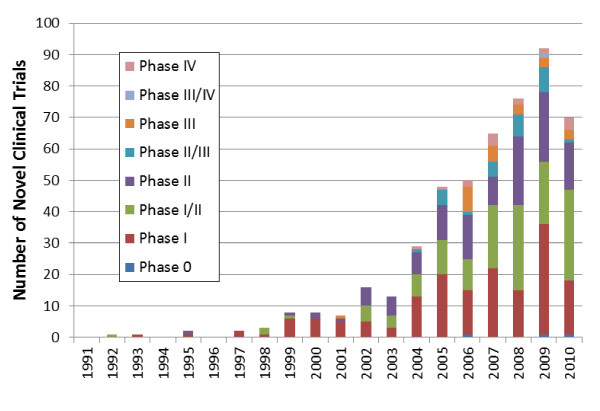
**Study phases of novel clinical trials**. Note that only 512 out of 639 clinical trials clearly indicated study phase. The drop in clinical trials in 2010 may be due to a lag in registration of clinical trials in databases by investigators.

**Figure 4:**
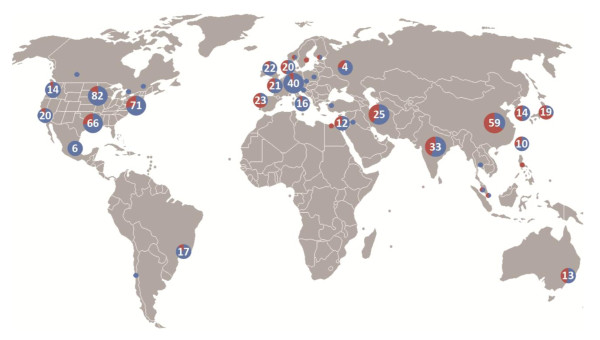
**Global map of innovative clinical trials representative of the future of regenerative medicine, indicating the proportion using mesenchymal stem cells (MSCs; in red)**. Numbers denote the total number of novel clinical trials in the region; small circles denote regions with one to three novel clinical trials.

In contrast, all CTs (n = 3,404) were dominated by use of adult SCs, primarily hematopoietic SCs, with some trials using umbilical cord blood derived SCs. There were an increasing number of trials using mesenchymal stem cells (MSCs) from 2007 (Figure 1B). The latter dominated our novel classification for CTs, most of which were in phases I and II (Figure 3). Figure 4 shows the global landscape for innovative CTs, with higher proportions using MSCs in Asia and the Middle East.

As of 2011, iPSCs had not entered CTs, and the best-known trial using hESCs, started in October 2010 by Geron for spinal cord injury, was halted in November 2011 after use in only four trial subjects. Three additional trials by Advanced Cell Technology (Santa Monica, CA, USA) began in July 2011: two using retinal pigment epithelial cells derived from hESCs for Stargardt's macular dystrophy and one for dry age-related macular degeneration [20]. CTs for cancers and graft-versus-host disease (GVHD), which composed most of the 'other diseases' category, dominated the SC CT landscape (Figure 2B). Nevertheless, there was a steady increase in CTs for cardiovascular disease beginning in 2001 and a small number of trials for neurological, ocular conditions and diabetes (note that the key word analysis for diabetes also captured CTs for associated morbidities such as diabetic foot). Our detailed examination of novel CTs was dominated by trials using MSCs and for conditions other than hematological cancers.

Our criteria for novel SC CTs resulted in the selection of 639 (19%) CTs that, if successful, would become the next generation of regenerative treatments. A total of 512 of these CTs clearly indicated study phase, and, of these, the majority were safety trials in phases I or II (Figure 4). Only 54 novel CTs were in phases II/III or III and 17 were in phases III/IV or IV (Table 1). The majority of clinical trials in phase III/IV (n = 11) used drug treatments to activate endothelial progenitor cells for therapeutic purposes in cardiovascular diseases (which may be associated with other diseases such as diabetes and ankylosing spondylitis). A further three advanced stage trials used stents to facilitate vascular repair by capturing endothelial progenitor cells in the stent and three trials addressed metabolic syndrome, critical limb ischemia, and breast deformities following a lumpectomy.

**Table 1:** Novel clinical trials (CTs) in phase II/III, phase III, phase II/IV or phase IV (n = 71) (note that phase II/IV and phase IV trials all fell in the drug treatments, stents or other categories (n = 17))

Clinical trial subject	No. of CTs	Country
Bone conditions	3	Iran, USA
Cardiovascular disease	29	Brazil, Canada, China, Finland, Germany, India, Iran, Italy, Mexico, The Netherlands, Russia, South Korea, UK, USA
Cartilage damage	2	Egypt, South Korea
Crohn's Disease	4	UK, USA
Diabetes	2	India, China
Multiple sclerosis	1	USA
Ocular surface disease	2	Malaysia, Thailand
Scleroderma	3	USA
Spinal cord injury	1	India
Drug treatments for stem cell mobilization	11	China, Germany, Italy, South Korea, Turkey, USA
Stents for capturing endothelial progenitor cells	3	China, The Netherlands, Poland
Other (mainly graft-versus-host disease)	3	India, Germany, UK

While the public and policymakers expect therapies for neurological conditions, there was limited activity in what may be considered novel application of SC therapies. Among the novel CTs, only 69 trials addressed neurological conditions (Table 2) and only 1 in our dataset, the Geron (Menlo Park, CA, USA) trial, used hESCs. The majority of trials used hematopoietic stem cells or MSCs. For example, clinical trials for multiple sclerosis used autologous transplantation of MSCs or hematopoietic SCs following chemotherapy to achieve immunomodulation and facilitate regeneration of the central nervous system. Two trials, being conducted in India, for Parkinson's disease used MSCs.

**Table 2:** Novel clinical trials (CTs) for neurological conditions (n = 69)

Clinical trial subject	No. of CTs	Country
Amyotrophic lateral sclerosis	7	China, Israel, Spain, USA
Cerebral palsy	3	Mexico, South Korea, USA
Cerebral palsy using MSCs	3	China, India
Diabetic neuropathy	3	Germany, Mexico, USA
Ischemic Stroke	18	Brazil, Canada, France, India, Malaysia, Russia, Spain, Taiwan, UK, USA, Russia
Multiple sclerosis	12	Canada, Israel, Spain, UK, USA
Neuronal ceroid lipfuscinosis	2	USA
Parkinson's Disease	2	India
Pelizaeus-Merzbacher Disease	1	USA
Spinal cord injury (non-hESC)	5	Egypt, India, USA
Spinal cord injury (hESC)	1	USA
Traumatic brain injury	3	Canada, India, USA
Other	10	Brazil, China, The Netherlands, South Korea, Thailand, USA

hESCs = human embryonic stem cells; MSCs = mesenchymal stem cells.

The majority of novel trials were publicly funded; however, some industry involvement was evident. Industry trials targeted both orphan and common diseases. Three phase I trials by StemCells Inc. (Newark, CA, USA) employed cultured allogeneic neural SCs (HuCNS-SC) to treat Pelizaeus-Merzbacher disease and neuronal ceroid lipfuscinosis. Ischemic stroke was the focus of significant industry involvement with 6 of 18 CTs sponsored by Stem Cell Therapeutics (Toronto, Canada), ReNeuron (Guildford, UK), and Stempeutics Research (Bangalore, India). The phase I trial by ReNeuron used a manufactured neural SC line (CTX cells) while the four Stem Cell Therapeutics trials attempted to use a proprietary drug (NTx-265) to promote neural regeneration. However, news reports indicated the lack of demonstrable effect in the latter CTs [21].

Of all the other diseases highlighted in the media, 208 CTs were for cardiovascular disease: 75% targeted the heart and 25% the peripheral vascular system. Almost all of these trials involved MSCs, mobilization of hematopoietic SCs, other purified bone marrow-derived SCs, or endothelial progenitor cells for regenerative and supportive purposes. There were 27 novel trials for diabetes mellitus, and these CTs attempted to treat both Type I and Type II diabetes using hematopoietic and MSCs from adipose tissue, umbilical cord blood, and bone marrow. Private sector companies were involved as collaborators or sponsors in eight of the trials, including Osiris Therapeutics (Columbia, MD, USA), Adistem (Hong Kong, China), Transition Therapeutics (Toronto, Canada), Cellonis Biotechnology (Beijing, China), and Genzyme (Cambridge, MA, USA).

## Discussion

Innovative biomedical technologies are prone to 'social bubbles' where categories of 'enthusiastic supporters weave a network of reinforcing feedbacks that lead to widespread endorsement and extraordinary commitment by those involved in the project' [22]. The enthusiasm of stakeholders such as researchers, the public, politicians, and funders in promoting optimistic timelines often masks the slow uptake of clinical applications and technologies within health systems, whether in biotechnology [23,24] in general or, more specifically, in genomics [25] or SC research. Our results show that there is a large gap between public expectations and clinical realities, with public representations focused on hESCs and on cures for neurological conditions. There is little awareness of the dominant clinical applications that use hematopoietic SCs and increasingly use MSCs. This leaves patients, desperately hoping for life-altering therapies, vulnerable to unscrupulous providers of unproven and expensive treatments. Indeed, there is a direct correlation between the numerous therapies for neurological conditions advertised by SC tourism clinic providers and media coverage dominated by neurological diseases as described in the study by Lau *et al. *[7].

### Why do therapies take so long to reach patients?

Stem cell biology is highly complex and variable; using cells as therapeutic agents bears few parallels to the use of small molecule drugs. This creates significant challenges for regulators developing the testing necessary to assure the quality of novel technologies and therapies, for businesses that require reproducible large scale production capacity, and for the health systems that need to find the funds for what will likely be personalized and expensive therapies. As an example, there are valid concerns about manufacturing consistency of cell cultures and the genetic stability of cell lines. Both hESCs and iPSCs have been shown to experience genetic and phenotypic drift in long-term *in vitro *culture. There are further concerns about iPSCs due to the degree to which differentiated adult cells must be manipulated to become pluripotent [26,27]. Given that there is a potential for late tumor formation resulting from inadvertent mutagenesis in the creation of SC products, prolonged monitoring of animals used in preclinical studies and long-term monitoring of CT participants will be required. SCs that are manipulated or manufactured or used in a non-homologous manner [28] require more stringent oversight to ensure the safety of the recipient.

Regulators are still developing systems specifically for complex cell therapies. The conventional preclinical regulatory studies for absorption, distribution, metabolism, excretion, and toxicity of drugs cannot be applied to cell-based therapies, in part because of an inability to track differentiation and migration of transplanted cells [15]. In contrast to drugs, SC based therapies may result in long-term engraftment, with amplification of the administered dose of SC and further growth and differentiation of the SC progeny. Regulators, in consultation with the SC research community, are attempting to develop a proportionate response which will balance patient safety yet will not stifle innovation in this field.

Developing or modifying regulatory pathways takes time, trust, and communication between regulators and the research community, all of which are undermined by hype [15,28] or public representations of SC research that do not reflect its current state and the realistic developmental timelines for specific diseases or conditions, as documented by this study. Even with cooperation between SC researchers and regulators, oversight may prove difficult in a translational environment that has gone global, as demonstrated in Figure 3. Significantly, our results show that CTs testing novel SC approaches are occurring beyond the borders of North America and Europe. Indeed, while there are SC trials for novel application increasingly being registered from sites in India, China, and Iran, and to a lesser extent in Brazil, Mexico and Malaysia, these may use SC with only rudimentary characterization or for situations with as yet poorly understood biological rationale; the very types of trials concerning to the ISSCR. Ethical issues and conflicts of interests are more likely to arise where inadequately developed regulatory and consent frameworks exist. This will be of special concern in an arena such as the complex regulatory environment for cell therapies that necessitate long-term monitoring and follow-up for participants.

Media reporting of therapeutic benefits, especially for neurological conditions, may exacerbate the 'therapeutic misconception' in all countries [29]. While our analysis demonstrates that most SC CTs are in phases I or II, participants may conflate such early stage safety trials with therapeutic benefit. This mistaken therapeutic belief, which undermines informed consent, may arise from poorly structured consent forms or from the descriptions of CTs in trial registries [30]. In reality, most therapies fail in phase I and II, and those that are proven both safe and effective take between US$200 million and US$1 billion in investment over a span of 10 to 15 years before they are approved and adopted by the medical community [31].

A further challenge highlighted in our results of public versus private support of trials is that commercial enterprise has been slow to move into the cell therapy arena, primarily because of the lack of easily defined business models [32]. Cell therapies are not drugs and require the development of new manufacturing and distribution systems. While a few allogeneic products with central manufacturing have been developed, most notably Carticel from Genzyme and Apligraf from Organogenesis, the closest model to many of the current CTs is hematopoietic SC transplantation (HSCT). HSCT is a high-cost, highly specialized treatment, requiring significant infrastructure and an interdisciplinary network of healthcare professionals [33,34]. Its global use is highly correlated with gross national income, healthcare expenditure and the availability of transplant teams. Given these system costs, the bar for therapeutic value, which compares the benefits of SC therapies with existing treatments, will be high. The model also belies the anticipated health system savings promised by proponents of SC research. These financial pressures, such as the scientific and regulatory issues, are absent from media reports and are hidden from public discourse, contributing to the disconnected state of current expectations.

## Conclusions

Given the research, regulatory, commercialization and health system hurdles involved in the clinical translation of SC research, it seems likely that patients and political supporters will become disappointed and disillusioned. In this environment, proponents need to make a concerted effort to temper claims. It is simply 'unfair to raise people's hopes to unrealistic levels when they and their families are desperate for treatments that will relieve their suffering and improve their health' [35]. Even though the field is highly promising, the road to the clinic is long and hard, and the technology may yet develop in unanticipated ways. For a field that, lacking significant private investment, is largely reliant on public support, there needs to be a more honest acknowledgement of the expected therapeutic benefits and the timelines to achieving them.

## Competing interests

The authors declare that they have no competing interests.

## Authors' contributions

TB and AH conceptualized the research question. AH, MH, MDL and MB obtained and analyzed data. AH and MH designed the CT coding methodology. TB, MDL and AH drafted the paper. AH and MH designed the CT coding methodology. All authors were involved in editing the paper and approved the final text.

## Authors' information

Dr Tania Bubela has a PhD from the University of Sydney in biology and a JD from the University of Alberta. She researches in the field of health law and policy as an Associate Professor, School of Public Health, University of Alberta. Dr Harry Atkins is a stem cell transplant physician at the Ottawa Hospital Research Institute and is currently the principal investigator of a clinical trial using hematopoietic stem cell transplantation for multiple sclerosis. Mark Bieber is a computer and data management specialist at the University of Alberta. Dr Hafez has an MB and a BCh from Cairo University, and an MHA from the University of Ottawa. At the time of this study, he was working as a research assistant with Dr Atkins, but is currently Medical Education Manager with Janssen Inc. Matthew D Li is a research assistant with Dr Bubela and, from 2012, a medical student at Stanford University.

## Pre-publication history

The pre-publication history for this paper can be accessed here:

http://www.biomedcentral.com/1741-7015/10/133/prepub
